# Treatment of hepatitis C virus infection with direct-acting antiviral agent-based regimens in Iranian patients with hereditary bleeding disorders

**DOI:** 10.1186/s12985-021-01659-0

**Published:** 2021-10-07

**Authors:** Heidar Sharafi, Bita Behnava, Alireza Azizi-saraji, Ali Namvar, Ali Anvar, Shima Salimi, Seyed Moayed Alavian

**Affiliations:** 1 Middle East Liver Diseases (MELD) Center, Tehran, Iran; 2 Iranian Comprehensive Haemophilia Care Centre, Tehran, Iran; 3 Baqiyatallah Research Center for Gastroenterology and Liver Diseases (BRCGL), Baqiyatallah University of Medical Sciences, Tehran, Iran

**Keywords:** Chronic hepatitis C, Direct-acting antiviral agents, Hereditary bleeding disorders, Sofosbuvir, Daclatasvir, Ledipasvir

## Abstract

**Background:**

Chronic hepatitis C (CHC) is one of the most important comorbidities in patients with hereditary bleeding disorders (HBD). The present study aimed at evaluating the effectiveness of direct-acting antiviral agent (DAA)-based interferon-free HCV antiviral regimens in patients with HBD.

**Patients and methods:**

The present study was performed on the patients with HBD and CHC between 2015 and 2019. Sofosbuvir-based interferon-free regimens with or without ribavirin were prescribed to treat HCV infection. The main endpoint of the study was to determine the sustained virologic response (SVR), assessed 12 weeks after the completion of treatment.

**Results:**

A total of 147 patients with a mean age of 41.1 years were enrolled in the study; 4.1% of them were co-infected with HIV, 25.2% had cirrhosis, and 76.9% of them were diagnosed with hemophilia A. HCV genotype-1 includes the largest number (68.1%) of patients. 46.3% of patients were treatment-naïve and others had a treatment history with interferon-based regimens. Out of 147 patients, 15 patients were lost to follow-up during treatment or for SVR evaluation or discontinued treatment. 132 subjects completed treatment and were evaluated for SVR, 12 weeks after the completion of treatment. All of the patients achieved SVR 12 (SVR rate: 100%, 95% CI 97.2–100%).

**Conclusion:**

Hepatitis C DAA-based regimens are the effective treatments for CHC in patients with HBD, regardless of the treatment modifiers such as previous treatment experience, cirrhosis, HIV co-infection, and HCV genotype.

## Introduction

Hereditary bleeding disorders (HBD) are heterogeneous disorders, the most common of which are hemophilia A and B. Due to the lack of effective blood and blood product screening before 1990, many patients with HBD were infected with the hepatitis C virus (HCV) [1]. A meta-analysis showed that 48% of Iranian hemophilic patients were infected with HCV that also varies 24–89% in different countries of the world [1, 2]. About 75–85% of patients who contracted HCV will develop chronic hepatitis C (CHC), which is associated with the increased risk of cirrhosis, end-stage liver diseases, and hepatocellular carcinoma [3]. HCV is also one of the leading causes of death in patients with hemophilia [4].

Before the 2010s, HBD patients with HCV infection were treated with pegylated interferon (Peg-IFN) plus ribavirin (RBV) [5]. Using Peg-IFN and RBV, the rate of sustained virologic response (SVR) varies from 29 to 79%, which is lower in HIV-infected or HCV genotype-1 patients than in others [6]. Moreover, due to the many side-effects of these drugs and their injectable nature, many patients abandoned therapy [7]. In recent years, with the introduction of direct-acting antiviral agents (DAAs), an evolution occurred in the treatment of HBD patients with HCV infection, so that the SVR rate increased to 98% following the use of DAAs [8]. Hence, with advances in HCV management, the World Health Organization (WHO) decided to plan for hepatitis C elimination by 2030 [9].

Patients with HBD are excluded from many studies evaluating the effectiveness of DAAs since they were considered a particular group of patients [3]. Therefore, it is of great importance to assess the effectiveness of DAAs for the treatment of HCV infection in these patients, since they constitute an important group of patients with CHC. Moreover, our knowledge about the effectiveness of DAA-based regimens for treating CHC in patients with HBD is limited.

Iran is one of the countries trying to eliminate HCV, so that with HCV Ab prevalence of 0.5% in the general population, it has the lowest prevalence of this virus in the Middle East, but the 9th place in the world in terms of the number of individuals living with hemophilia [10–12]. According to Iran's national guidelines, the administration of DAAs, including sofosbuvir (SOF), daclatasvir (DCV), ledipasvir (LDV), and most recently, velpatasvir (VEL) with or without RBV, for 12 or 24 weeks is recommended for the treatment of HCV infection depending on the previous treatment experience, co-infection with HIV, and HCV genotype [10–13].

The present study aimed at evaluating the rate of SVR in Iranian patients with HBD and CHC treated with SOF-based IFN-free regimens. Efficient hepatitis C treatment uptake and linkage to care among patients with HBD, one of the important groups of patients with HCV infection, is of great importance considering the global plan of WHO to eliminate hepatitis C by 2030.

## Patients and methods

### Study population and design

The current bicentric, retrospective study was performed on patients selected from two centers of Middle-East Liver Diseases (MELD) center (Tehran, Iran) and Iranian Comprehensive Hemophilia Care Center (ICHCC) (Tehran, Iran). Patients with HBD aged above 18 years who had HCV infection for at least six months and a positive result for HCV RNA by RT-PCR undergoing CHC therapy with SOF-based IFN-free regimens from 2015 to 2019 in the two above-mentioned centers were enrolled in this study. Besides, patients co-infected with HIV, the ones with cirrhosis, and patients with a history of IFN-based therapy were also included. Patients who had previously responded to treatments such as IFN or other available options and were not treated with DAA-based regimens were excluded. Two researchers extracted the required information by evaluating patients' electronic records at MELD Center and paper-based records at ICHCC.

### Treatment regimens

In Iran, SOF/DCV, SOF/LDV, and SOF/VEL were available as generic forms, and patients undergo a 12- or 24-week therapy with or without RBV, based on HCV genotype, previous treatment experience, and having cirrhosis according to national and international guidelines [10, 13–15]. From 2015 to 2019, the standard of care for the management of CHC had updated regularly but the common treatment discipline in these years was that patients with cirrhosis, previous treatment experience, or HCV genotype-3 need a more intensified treatment. Moreover, case-by-case clinical decisions outside the therapeutic guidelines were made by the treating physician to provide the patients desirable benefits [10–13]. HCV DAA-based regimens can be used alongside anti-HIV regimens with consideration of drug–drug interactions. In this regard, DCV dose adjustment is needed considering the anti-HIV regimen [16]. Sofosbuvir interaction with anti-epileptic drugs (such as phenytoin, phenobarbital, and carbamazepine) and amiodarone was considered before starting treatment with DAA. In cases with drug–drug interactions, we consulted with their specialists to switch their treatments to remove the proposed drug–drug interaction.

### Liver fibrosis and laboratory assessments

Complete blood cell count, liver enzymes, and HCV RNA using RT-PCR were performed before starting treatment, four weeks after initiation of treatment, and 12 weeks after the completion of treatment. Liver stiffness measurement (LSM) was also performed to detect cirrhosis before starting treatment. However, in cases where clinical (e.g., esophageal varices) or paraclinical (e.g., ultrasound findings) pieces of evidence were strongly in favor of cirrhosis, the LSM was ignored.

### Endpoints and definitions

A patient with no history of treatment for CHC was called treatment-naïve. If IFN or peg-IFN is used in the previous treatment, the treatment history is considered as IFN-based treatment history, and if the previous treatment was IFN-free, using a combination of DAAs, it was considered as DAA-based treatment history. Patients who discontinued treatment, who did not refer during treatment, or did not test for HCV RNA, 12 weeks after the completion of treatment were called treatment discontinuation, lost to follow-up on treatment, and lost to follow-up for SVR evaluation, respectively. Moreover, conditions with undetectable results for HCV RNA four weeks after initiation of treatment and 12 weeks after the completion of treatment were called rapid virologic response (RVR) and SVR, respectively. Cases that do not reach SVR were considered a virologic treatment failure.

Our primary endpoint in the present study was to evaluate the SVR rate in patients with HBD and CHC treated with SOF-based IFN-free regimens.

### Statistical analysis

All data were transferred into SPSS version 23.0 (IBM Corp.). Data obtained from quantitative variables with normal distribution were expressed as mean ± standard deviation (SD) and those from variables that deviated from normal distribution as median and interquartile range. The Kolmogorov–Smirnov test was used to determine the normal distribution of variables. Qualitative variables were also expressed as absolute value and percentage. HCV RNA level was also expressed in logarithmic form.

## Results

### Baseline characteristics

A total of 147 patients with HBD and CHC started treatment with DAA-based regimens. The mean (± SD) age of 147 patients was 41.1 (± 11.6) years, and 94.6% were male. Six (4.1%) patients were co-infected with HIV, and 37 (25.2%) had cirrhosis (five with decompensated cirrhosis). HCV genotypes-1 and -3 with 98 (68.1%) and 30 (20.8%) patients had the highest frequency, respectively. Of these 147 patients, 113 had hemophilia A (HA), 19 had hemophilia B (HB), eight had von Willebrand disease (VWD), one had Glanzmann disorder, and six had other disorders. The median values of hemoglobin (Hb), AST, and ALT before the treatment were 15.7 g/dL, 38 IU/L, and 42 IU/L, respectively. The mean HCV RNA level at baseline was 6.0 log_10_ IU/mL (Table 1). The anti-HIV regimens in the 6 patients with HIV-HCV co-infection were zidovudine + lamivudine + efavirenz (two patients), tenofovir + lamivudine + efavirenz (two patients), lopinavir/ritonavir + efavirenz (one patient), and lopinavir/ritonavir + lamivudine + tenofovir (one patient). Five patients with HIV-HCV co-infection were treated with SOF/LDV ± RBV that did not need a dose adjustment. However, one patient with HIV-HCV co-infection was subjected to DCV dose adjustment from standard 60 mg dose to 90 mg dose. The baseline characteristics except for the type of HBD (*P* < 0.05) were not different between patients who were evaluated for SVR and patients with treatment discontinuation or loss to follow-up (Table 1).

**Table 1:** Baseline characteristics of patients

Characteristics	All patients (N = 147)	Patients who were evaluated for SVR (N = 132)	Patients with treatment discontinuation or loss to follow-up (N = 15)
Age*, mean ± SD (years)	41.1 ± 11.6	41.2 ± 11.9	40.1 ± 8.7
Gender, n (%)			
Male	139 (94.6%)	126 (95.5%)	13 (86.7%)
Female	8 (5.4%)	6 (4.5%)	2 (13.3%)
Hereditary bleeding disorder types, n (%)			
Hemophilia A	113 (76.9%)	106 (80.3%)	7 (46.7%)
Hemophilia B	19 (12.9%)	14 (10.6%)	5 (33.3%)
VWD	8 (5.4%)	6 (4.5%)	2 (13.3%)
Glanzmann	1 (0.7%)	1 (0.8%)	0 (0%)
Other disorders	6 (4.1%)	5 (3.8%)	1 (6.7%)
HIV co-infection, n (%)	6 (4.1%)	5 (3.8%)	1 (6.7%)
HBV co-infection, n (%)	0 (0%)	0 (0%)	0 (0%)
Platelet count**, mean ± SD (10^9^/L)	200.0 ± 59.3	197.8 ± 59.4	219.8 ± 56.6
Hemoglobin**, Median (IQR) (g/dL)	15.7 (14.3–16.9)	15.7 (14.4–16.9)	15.3 (11.1–17.3)
AST**, Median (IQR) (IU/L)	38 (27–55)	38 (27–55)	38 (32–76)
ALT**, Median (IQR) (IU/L)	42 (27–70)	42 (30–70)	46 (19–72.5)
HCV RNA*, mean ± SD (log_10_ IU/mL)	6.0 ± 0.8	5.9 ± 0.8	6.2 ± 0.5
Liver stiffness measurement (Metavir score)*, n (%)			
F0–F2	75 (54%)	66 (52.8%)	9 (64.3%)
F2–F4	33 (23.7%)	31 (24.8%)	2 (14.3%)
F4	31 (22.3%)	28 (22.4%)	3 (21.4%)
Cirrhosis, n (%)			
Non-cirrhotic	110 (74.8%)	99 (75%)	11 (73.3%)
Compensated cirrhosis	32 (21.8%)	29 (22.0%)	3 (20%)
Decompensated cirrhosis	5 (3.4%)	4 (3.0%)	1 (6.7%)
HCV genotype*, n (%)			
1	98 (68.1%)	89 (68.5%)	9 (64.3%)
2	2 (1.4%)	2 (1.5%)	0 (0%)
3	30 (20.8%)	27 (20.8%)	3 (21.4%)
4	4 (2.8%)	4 (3.1%)	0 (0%)
Mix	10 (6.9%)	8 (6.1%)	2 (14.3%)
Previous treatment experience, n (%)			
Treatment naïve	68 (46.3%)	61 (46.2%)	7 (46.7%)
Interferon-experienced	79 (53.7%)	71 (53.8%)	8 (53.3%)
DAA-experienced	0 (0%)	0 (0%)	0 (0%)

*SD* standard deviation, *IQR* interquartile range, *DAA* direct-acting antiviral agent, *VWD* Von Willebrand disease, *SVR* sustained virologic response, *n* number, *HIV* human immunodeficiency virus, *HBV* hepatitis B virus, *ALT* alanine aminotransferase, *AST* aspartate aminotransferase

^*^Data were missed in less than 10% of patients

^**^Data were missed in more than 10% of patients

### Antiviral response

**Fig. 1:**
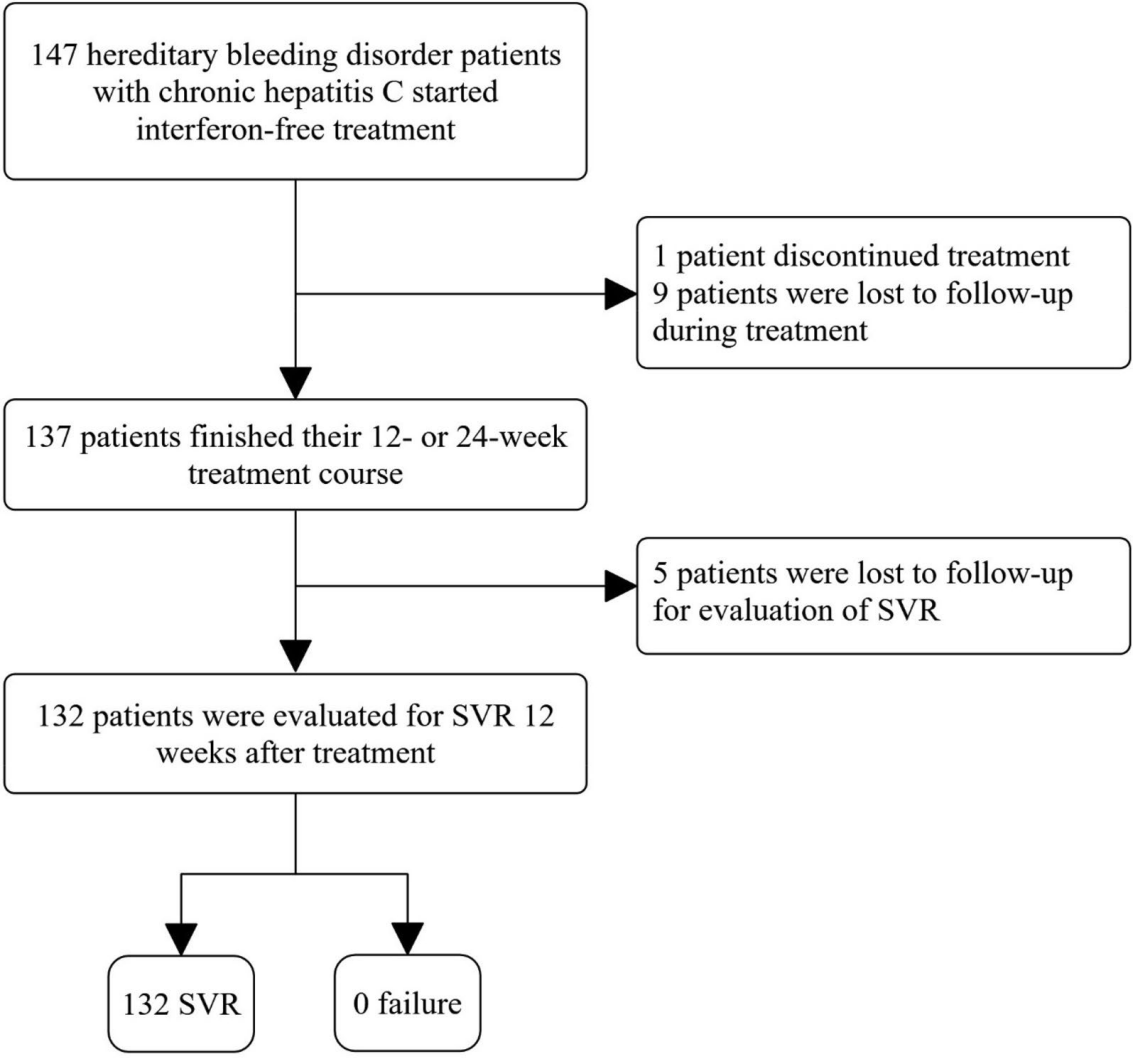
Flowchart of hepatitis C treatment

Treatment with DAA-based regimens was started for 147 patients with HBD and CHC. One patient discontinued the treatment arbitrarily after one month, with no side effects. Moreover, 9 patients did not refer during the treatment course, and it was not clear whether they completed the treatment or not. Five patients completed the treatment course but did not refer 12 weeks after treatment completion to determine SVR. The remaining 132 patients completed the treatment and were available for the evaluation of SVR (Fig. 1). Of the 147 patients who received DAA-based treatment, SOF/LDV + RBV was the most common regimen administered to 53 (36.1%) patients. SOF/LDV and SOF/DCV ± RBV regimens were other common regimens in this study. The regimens, treatment courses (12 or 24 weeks), and RVR and SVR rates are shown in Table 2. Regardless of HCV genotype, therapeutic regimen, HIV co-infection, liver disease status, and HCV RNA level at baseline, the HCV RNA was undetectable in all the 132 patients, 12 weeks after treatment completion (SVR rate: 100%, 95% CI 97.2–100%). Of these 132 patients, HCV RNA was evaluated on blood samples of 100 patients four weeks after the treatment initiation, of which only six were detectable, who finally achieved SVR. The characteristics of these six patients that didn’t achieve RVR are shown in Table 3. Of the 15 patients whose SVR status was unknown due to the reasons stated, HCV RNA detection was performed on the blood samples of nine of them, four weeks after the treatment initiation, which all were undetectable. Therefore, the overall RVR rate was 94.5% (95 CI 88.4–98.0%) in the present study (Table 2).

**Table 2:** Treatment regimens and antiviral response

Regimen	All patients (N = 147)	Patients who were evaluated for SVR (N = 132)	Patients with treatment discontinuation or loss to follow-up (N = 15)	Virologic responses			
				RVR (N = 109)		SVR (N = 132)	
				Rate (%)	95% CI of %*	Rate (%)	95% CI of %*
SOF/LDV 12 W	32 (21.8%)	30 (22.7%)	2 (13.3%)	15/16 (93.8%)	69.8–99.8%	30/30 (100%)	88.4–100%
SOF/LDV 24 W	9 (6.1%)	7 (5.3%)	2 (13.3%)	9/9 (100%)	66.4–100%	7/7 (100%)	59.0–100%
SOF/LDV + RBV 12 W	46 (31.3%)	42 (31.8%)	4 (26.6%)	39/41 (95.1%)	83.5–99.4%	42/42 (100%)	91.6–100%
SOF/LDV + RBV 24 W	7 (4.8%)	6 (4.5%)	1 (6.7%)	3/6 (50%)	11.8–88.2%	6/6 (100%)	54.1–100%
SOF/DCV 12 W	22 (15%)	20 (15.1%)	2 (13.3%)	14/14 (100%)	76.9–100%	20/20 (100%)	83.2–100%
SOF/DCV 24 W	4 (2.7%)	3 (2.3%)	1 (6.7%)	2/2 (100%)	15.8–100%	3/3 (100%)	29.2–100%
SOF/DCV + RBV 12 W	5 (3.4%)	4 (3.1%)	1 (6.7%)	3/3 (100%)	29.2–100%	4/4 (100%)	39.8–100%
SOF/DCV + RBV 24 W	13 (8.8%)	12 (9.1%)	1 (6.7%)	13/13 (100%)	75.3–100%	12/12 (100%)	73.5–100%
SOF/VEL 12 W	7 (4.7%)	7 (5.3%)	0 (0%)	4/4 (100%)	39.8–100%	7/7 (100%)	59.0–100%
SOF/VEL 24 W	1 (0.7%)	0 (0%)	1 (6.7%)	NA	NA	NA	NA
SOF + RBV 24 W	1 (0.7%)	1 (0.8%)	0 (0%)	1/1 (100%)	2.5–100%	1/1 (100%)	2.5–100%
Total	147 (100%)	132 (100%)	15 (100%)	103/109 (94.5%)	88.4–98.0%	132/132 (100%)	97.2–100%

*SOF* sofosbuvir, *LDV* ledipasvir, *DCV* daclatasvir, *VEL* velpatasvir, *RBV* ribavirin, *W* weeks, *RVR* rapid virologic response, *SVR* sustained virologic response, *NA* not available, *CI* confidence interval, *N* number

^*^Binomial exact calculation

**Table 3:** Characteristics of patients who did not achieve rapid virologic response

No	Age	Sex	HBD	Regimen	Liver disease	HCV genotype	Previous treatment experience	Baseline HCV RNA (log_10_ IU/mL)	Week 4 HCV RNA (log_10_ IU/mL)	SVR12 status
2	41	Male	HA	SOF/LDV + RBV 24 W	Non-cirrhotic	4	Naïve	6.94	2.24	SVR
5	31	Male	HB	SOF/LDV + RBV 24 W	Non-cirrhotic	1	IFN-based	5.77	3.68	SVR
36	49	Male	HA	SOF/LDV + RBV 12 W	Non-cirrhotic	1	Naïve	5.91	2.53	SVR
59	39	Male	HB	SOF/LDV + RBV 24 W	Compensated cirrhosis	1	IFN-based	6.64	2.62	SVR
122	34	Female	VWD	SOF/LDV + RBV 12 W	Non-cirrhotic	1	Naïve	6.21	2.12	SVR
130	32	Male	HA	SOF/LDV 12 W	Non-cirrhotic	1	IFN-based	7.04	5.59	SVR

*HBD* hereditary bleeding disorder, *HA* hemophilia A, *HB* hemophilia B, *VWD* Von Willebrand disease, *SOF* sofosbuvir, *LDV* ledipasvir, *RBV* ribavirin, *IFN* interferon, *SVR* sustained virologic response, *W* weeks

### Treatment side-effects

Among 132 patients with follow-up, none expired during treatment or 12 weeks after treatment termination, and no significant side-effect causing treatment discontinuation was observed. In three patients that treatment was commenced with RBV, due to gastrointestinal bleeding, RBV was removed from the treatment course. Despite the removal of RBV from treatment, all of these three patients achieved RVR and SVR. Week-4 Hb reduction was more prominent in patients treated with RBV (38.1% with > 2 g/dL Hb decline) than those who did not receive RBV (3.2% with > 2 g/dL Hb decline).

## Discussion

Patients with HBD and CHC were considered as difficult-to-treat due to co-infection with HIV, more advanced liver diseases due to prolonged HCV infection, predominance of the male gender, and predominance of HCV genotype-1, all are SVR-reducing parameters in patients undergoing peg-IFN therapy [6, 17, 18]. An evolution occurred in the treatment of patients with CHC following the introduction of DAAs. Subsequently, in May 2016, the hepatitis C elimination plan was described by WHO to reduce active cases of HCV infection by 80% and a 65% reduction in HCV-related death by 2030 [9]. In 2017, The European Association for the Study of the Liver (EASL) defined a new term called micro-elimination. In the micro-elimination approach, patients were divided into smaller groups to get prevention, care, and treatment faster and more effectively. Incarcerated individuals, HBD patients, and people who inject drugs are examples of HCV micro-elimination subgroups. This method will be useful to get the WHO program to eliminate HCV [19].

European Association for the Study of the Liver, American Association for the Study of the Liver Diseases (AASLD), and Infectious Diseases Society of America (IDSA) recommended therapeutic regimens consisting of SOF + DCV, SOF/LDV, SOF/VEL, SOF/VEL/VOX (voxilaprevir), glecaprevir/pibrentasvir (GLE/PIB), elbasvir/grazoprevir (EBR/GZR), and SOF + EBR/GZR with or without RBV for 8, 12 or 24 weeks [16, 20]. The SVR rate reaches 90–99% by taking these regimens [21–25]. Sofosbuvir, an NS5B polymerase inhibitor, plus an NS5A inhibitor, available as LDV, DCV, and VEL, is prescribed in Iran to treat CHC for 12 or 24 weeks with or without RBV [13]. The SVR rates yielded following these regimens were reported 96.7–100% in Iran [26–28].

Nine studies from Belgium, the USA, Japan, South Korea, New Zealand, Italy, China, and Portugal thus far measured SVR rate in patients with HBD and CHC; except two studies from the United States and Italy, as well as the present study, their sample sizes were less than 100 [3, 7, 17, 29–34]. The information of these studies is summarized in Table 4.

**Table 4:** Literature review of studies on the efficacy of IFN-free regimens in hereditary bleeding disorder patients with HCV infection

Author	Study date	Study location	Sample size, n	Anti-HCV regimen, %	HCV genotype, %	Treatment naïve, %	HIV co-infection, %	Cirrhosis, %	SVR
Fransen et al. [29]	2019	Belgium	85	SOF/LDV: 2.4%	NA*	72.6	NA*	12	77/84 (91.6%)
				SOF/VEL: 16.4%					
				SOF + SIM: 5.9%					
				SOF + DCV: 12.9%					
				OBV/r/PTV ± DSV: 20.0%					
				EBR/GZR: 21.2%					
				GLE/PIB: 12.9%					
				Others**: 8.2%					
Walsh et al. [3]	2017	US	120	SOF/LDV: 86.7%	1: 0.8%	60	21.7%	30.8	118/120 (98.3%)
				SOF + RBV: 13.3%	1a: 59.2%				
					1b: 25.9%				
					2: 8.3%				
					3: 5%				
					4: 0.8%				
Nagao et al. [31]	2017	Japan	43	SOF/LDV: 100%	1a: 58.1%	58.1	46.5%	20.9	41/43 (95.3%)
					1b: 27.9%				
					1a + 2b: 2.3%				
					4: 11.7%				
Lee et al. [7]	2017	Korea	30	DCV + ASV: 60%	1b: 70%	70	NR	13.3	28/30 (93.3%)
				LDV/SOF: 26.7%	1a: 16.7%				
				SOF + RBV: 13.3%	2a/2b: 13.3%				
Stedman et al. [32]	2016	New Zealand	14	SOF/LDV + RBV: 100%	1a: 71%	79	0%	7	14/14 (100%)
					1b: 29%				
Mancuso et al. [17]	2020	Italy	200	SOF/VEL: 22.5%	1a: 63%	54	20%	28	198/200 (99%)
				GLE/PIB: 20%	1b: 15%				
				SOF/LDV: 11.5%	1: 1%				
				SOF/LDV + RBV: 10%	2: 12%				
				SOF + SIM: 6.5%	3: 7%				
				GZR/EBR: 6.5%	4: 3%				
				DSV/PTV/OBV/r: 5.5%					
				DSV/PTV/OBV/r + RBV: 5%					
				SOF + DCV + RBV: 4%					
				DCV + RBV: 2.5%					
				SOF + DCV: 2%					
				SOF + RBV: 1.5%					
				SOF/VEL + RBV: 1.5%					
				SOF + SIM + RBV: 1%					
Xiao et al. [34]	2019	China	12	SOF + DCV: 66.7%	1b: 75%	41.7	100%	33	12/12 (100%)
				SOF/VEL: 16.7%	2i: 16.7%				
				SOF + RBV: 8.3%	3: 8.3%				
				DCV + ASV: 8.3%					
Uemura et al. [33]	2017	Japan	27	SOF/LDV: 85.2%	1a: 15%	33	100%	41	27/27 (100%)
				SOF + RBV: 3.7%	1b: 59%				
				SOF + DCV: 11.1%	1: 7%				
					2a: 4%				
					3a: 11%				
					4a: 4%				
Guedes et al. [30]	2020	Portugal	16	SOF/LDV: 75%	1a: 40%	12.5	NR	62.5	16/16 (100%)
				SOF/LDV + RBV: 6.25%	1b:46.6%				
				SOF + RBV: 6.25%	2: 6.7%				
				OBV/PTV/r + DSV: 6.25%	3a: 6.7%				
				EBR/GZR: 6.25%					
Current study	2020	Iran	147	SOF/LDV: 27.9%	1: 68.1%	46.3	4.1%	25.2	132/132 (100%)
				SOF/LDV + RBV: 36.1%	2: 1.4%				
				SOF/DCV: 17.7%	3: 20.8%				
				SOF/DCV + RBV: 12.2%	4: 2.8%				
				SOF/VEL: 5.4%	Mix: 6.9%				
				SOF + RBV: 0.7%					

*NR* not reported, *NA* not available, *DSV* dasabuvir, *EBR* elbasvir, *GLE* glecaprevir, *GZR* grazoprevir, *LDV* ledipasvir, *OBV* ombitasvir, *VEL* velpatasvir, *VOX* voxilaprevir, *DCV* daclatasvir, *ASV* asunaprevir, *SIM* simprevir, *PTV* paritaprevir, *PIB* pibrenastavir, *r* ritonavir, *SVR* sustained virologic response, *HIV* human immunodeficiency virus

^*^The HCV genotyping and HIV co-infection data were reported for total cases, not patients who received DAAs

^**^Not mentioned in the article

Fransen et al. [29] performed a study in Belgium on 85 patients with HBD and CHC treated with DAAs. Of these 85 patients, the SVR rate was measured in 84, and 77 (91.6%) achieved SVR. All the seven patients not reaching SVR were HCV genotype-1b, treatment-naïve, and non-cirrhotic. Five patients were treated with EBR/GZR, one with ombitasvir (OBV)/ritonavir (r)/paritaprevir (PTV) ± dasabuvir (DSV), and one with SOF/VEL. Walsh et al. [3] conducted a study on 120 patients with HBD and CHC treated with DAAs. The SOF/LDV regimen was administered to 104 patients with HCV genotype-1 or -4. 10 patients with HCV genotype-2 and six patients with HCV genotype-3 were treated with SOF + RBV. Among all 104 patients receiving SOF/LDV, except one who was lost to follow-up, others achieved SVR. Moreover, all 10 patients with HCV genotypes-2 and five (83%) patients with HCV genotype-3 achieved SVR. The study by Nagao et al. [31] in Japan treated 43 patients with hemophilia and CHC using SOF/LDV for 12 weeks. In this study, 96% of the patients achieved SVR, and the SVR rate was significantly different between cirrhotic and non-cirrhotic patients (78% for cirrhotic and 100% for non-cirrhotic patients, *P* value = 0.005). Mancuso et al. [17] conducted a study in Italy on 200 patients, 61% of whom underwent the SOF-based treatment and 20% treated with GLE/PIB regimen. Only two patients didn’t achieve SVR that both were HIV-positive. In the above-mentioned study, the SVR rate of HIV-negative patients was significantly higher than that of HIV-positive ones (100% vs. 95%; *P* value = 0.04).

In the present study, 147 patients with HBD and CHC underwent SOF/LDV ± RBV, SOF/DCV ± RBV, SOF/VEL, and SOF + RBV regimens. 132 patients completed the treatment course, their SVR rate was measured, and all achieved SVR. The present study, following the study by Mancuso et al. [17], had the largest sample size with an SVR rate of 100%, which was similar to those of other studies (91.6–100%). While 94.5% of patients achieved RVR, all those without RVR had undetectable HCV RNA, 12 weeks after treatment termination (SVR). This confirms that on-treatment response (RVR) has no or little impact on treatment success (SVR) as proposed in a study by Kowdley et al. [35].

The pandemics of coronavirus disease 2019 (COVID-19) impacted the containment of other infectious diseases such as HCV care and elimination in Iran and worldwide. With the ongoing condition, there is a great concern regarding the achievement of WHO elimination goals [36]. Moreover, initiating HCV antiviral therapy in patients with current COVID-19 infection is not warranted [37].

The present study had some strengthens over the past studies such as a larger sample size in comparison to most of the previous studies. Moreover, the drugs were used in this study were generics that showed the same efficacy as those of brand-name drugs. The limitations of the current study were its retrospective design and evaluation of only the therapeutic regimens (SOF-based regimens) available in Iran.

## Conclusion

Our study showed that DAAs are highly effective in HBD patients for the treatment of HCV infection, irrespective of the treatment modifiers such as previous treatment experience, cirrhosis, HIV co-infection, and HCV genotype. These results demonstrated that we can take steps toward HCV micro-elimination in HBD patients in line with the WHO HCV elimination program in 2030.

## Data Availability

The datasets used and/or analyzed during the current study are available from the corresponding author on reasonable request.

## Abbreviations

CHC: Chronic hepatitis C
HBD: Hereditary bleeding disorders
DAA: Direct-acting antiviral agent
SVR: Sustained virologic response
HCV: Hepatitis C virus
RBV: Ribavirin
SOF: Sofosbuvir
DCV: Daclatasvir
LDV: Ledipasvir
VEL: Velpatasvir
RVR: Rapid virologic response
EASL: European association for the study of the liver
AASLD: American association for the study of the liver diseases
IDSA: Infectious diseases society of America
VOX: Voxilaprevir
GLE/PIB: Glecaprevir/pibrentasvir
EBR/GZR: Elbasvir/grazoprevir
OBV: Ombitasvir
r: Ritonavir
PTV: Paritaprevir
DSV: Dasabuvir
